# Factors Influencing Personalized Management of Vestibular Schwannoma: A Systematic Review

**DOI:** 10.3390/jpm12101616

**Published:** 2022-09-30

**Authors:** Bruno Sergi, Stefano Settimi, Gaia Federici, Costanza Galloni, Carla Cantaffa, Eugenio De Corso, Daniela Lucidi

**Affiliations:** 1 Department of Head, Neck and Sensory Organs, Università Cattolica del Sacro Cuore, 00168 Rome, Italy; 2 Unit of Otorhinolaryngology-Head and Neck Surgery, Fondazione Policlinico Universitario A. Gemelli IRCCS, 00168 Rome, Italy; 3 Department of Otolaryngology-Head and Neck Surgery, University Hospital of Modena, University of Modena and Reggio Emilia, 41125 Modena, Italy

**Keywords:** vestibular schwannoma, acoustic neuroma, translabyrinthine, retrosigmoid, middle cranial fossa, stereotactic radiotherapy, radiosurgery, gamma knife, quality of life

## Abstract

Management of vestibular schwannoma (VS) is a complex process aimed at identifying a clinical indication for fractionated stereotactic radiotherapy (sRT) or microsurgical resection or wait and scan (WS). The aim of the review was to clarify which patient and tumor parameters may lead to different therapeutic choices, with a view to a personalized VS approach. A systematic review according to Preferred Reporting Items for Systematic Review and Meta-Analysis criteria was conducted between February and March 2022. The authors defined six parameters that seemed to influence decision-making in VS management: 1-incidental VS; 2-tumor size; 3-tumor regrowth after sRT; 4-subtotal resection; 5-patients’ age; 6-symptoms. The initial search yielded 3532 articles, and finally, 812 articles were included. Through a qualitative synthesis of the included studies, management strategies were evaluated and discussed. An individualized proposal of procedures is preferable as compared to a single gold-standard approach in VS decision-making. The most significant factors that need to be considered when dealing with a VS diagnosis are age, tumor size and hearing preservation issues.

## 1. Introduction

Vestibular schwannomas (VS) account for 8%–10% of all intracranial neoplasms and are the most common tumors of the cerebellopontine angle [1]. Current management strategies vary considerably across centers and countries and decision-making has progressively become more complex [2,3]. Tumor parameters, including initial size and interval growth on serial imaging (often >2 mm between images), are commonly associated with the decision for treatment. Major advancements have been made in VS therapy: fractionated stereotactic radiotherapy or radiosurgery (sRT) provides a treatment option, alternative to the microsurgical resection in selected cases. Microsurgery, however, remains the mainstay of treatment for large tumors, the main approaches being the translabyrinthine and the retrosigmoid ones. Retrospective studies of large cohorts (up to 8330 patients collected on a 7-year US registry analysis) demonstrated that 48–59% of patients underwent microsurgery and 21–24% underwent radiotherapy, with surgical resection correlating with younger age and larger tumor size [4,5,6].

Increasing interest in quality of life (QoL) measurements arose in VS treatment in the past decade [7,8]. Several reports indicated that surgical intervention for VS has a significant impact on social functioning. Most VS patients have, in fact, minimal preoperative disability and treatment of VS is aimed at dealing with the disease, rather than patient symptoms per se. Adequate counseling is necessary and it must give realistic expectations. Although several cornerstones of diagnosis and therapy are shared among different centers, there are still controversies related to the characteristics of tumors and patients, as well as institutional preferences. Personalized medicine is a medical model that separates patients into different groups, with final strategies being tailored to the individual patient, based on their predicted response. The aim of the present review was to clarify which patient and tumor’s parameters may lead to different therapeutic choices, with a view to a personalized approach.

## 2. Materials and Methods

This review was conducted in accordance with the Preferred Reporting Items for Systematic Review and Meta-Analysis (PRISMA) process to identify published clinical articles regarding VS management. Manuscripts were screened by MEDLINE database, Cochrane review, LILACS, Web of Science and Google Scholar. Parentheses and Boolean operators (AND, OR) were applied to create conjunctions. The search was performed between February and March 2022 based on MeSH terms, as follows: [(acoustic schwannoma[MeSH Terms]) OR (acoustic neurinoma[MeSH Terms]) OR (acoustic neuroma[MeSH Terms])) AND ((surgical[MeSH Terms]) OR (surgery[MeSH Terms]) OR (radiotherapy[MeSH Terms]) OR (cyberknife radiosurgery[MeSH Terms]) OR (gamma knife radiosurgery[MeSH Terms]) OR (radiosurgery, stereotactic[MeSH Terms]) OR (retrosigmoid[All Fields]) OR (translabyrinthine[All Fields]) OR (fossa, middle cranial[MeSH Terms]) OR (observation[MeSH Terms])].

In the first screening, authors independently read the titles and abstracts of all articles performing the first selection, being as inclusive as possible. Any disagreements were resolved by consensus. In the second phase, the full articles were collected for the analysis, based on the following exclusion criteria: papers with no full text available, those written in languages other than English, Italian, French and Spanish, those regarding Neurofibromatosis 1-2 (NF 1-2) or other histological entities different from Schwannoma and affecting the internal auditory canal (IAC), those regarding Schwannomas of the seventh cranial nerves, case reports, systematic reviews, metanalysis, editorial letters, anatomical studies, surgical/technical notes, basic sciences and animal model studies. Moreover, only studies published from January 2001 to April 2022 were screened.

We excluded all the articles that did not meet the inclusion criteria or deal directly with the issue investigated. Additional studies were manually identified from the reference lists of retrieved literature. The authors extracted data from included articles using a standardized template and collected them into a computerized database. The authors, through a qualitative synthesis of the included studies, defined six parameters that seemed to influence decision-making in VS management, as follows: 1-incidental VS; 2-tumor size; 3-tumor regrowth after sRT; 4-subtotal resection; 5-patients’ age; 6-symptoms. Management strategies according to these issues were carefully evaluated and discussed.

## 3. Results and Discussion

**Figure 1:**
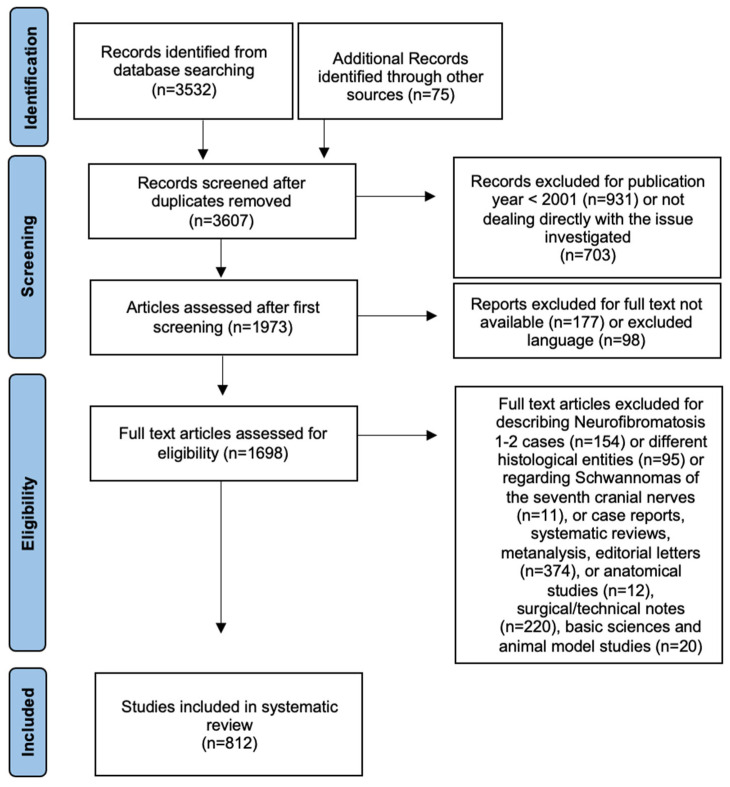
Flowchart of article search and selection according to the PRISMA criteria.

In total, our search yielded 3532 articles. A further manual check of the references included in the articles was performed, adding 75 articles. We excluded 703 articles for not dealing directly with the investigated issue, 931 articles for publication year < 2001, 177 records for full text not available, and 98 for language different than the included ones. Finally, 1698 full text articles were assessed for eligibility and 154 of those were excluded for describing NF 1–2 cases, 95 for histologic features different from Schwannoma, 11 for describing Schwannomas of the seventh cranial nerves and 626 for article type different from the included ones. The details of the systematic search are shown in Figure 1.

### 3.1. Incidental VS

The estimated incidence of incidental VS is around 0.2–0.3%, while it is higher in autoptical studies (around 1–2.4%) [9]. A completely asymptomatic VS found in a brain scanning performed for other reasons represents a clinical challenge [10]. On one hand, several studies proved that small, asymptomatic VS do not usually tend to grow, compared with larger and symptomatic ones, as described by Carlson et al. on a 38-patients’ cohort [10]. On the other hand, the best surgical results in terms of both facial function and hearing preservation are obtained in the population affected by small, asymptomatic VS, as demonstrated on a subset of 153 patients operated on by retrosigmoid approach [11]. The growth rate and time to treatment does not seem to differ between asymptomatic small VS versus symptomatic and larger ones, The survival-free tumor growth/treatment was 54% after 5 years. Despite this growth, very few patients experienced new symptoms. Moreover, as discussed below in the text, there is a paucity of prognostic factors that can predict growth and symptoms progression [10].

### 3.2. Tumor Size

Koos classification includes Grade I or intracanalicular tumor; Grade II orsmall tumor protruding at CPA, up to 20 mm; Grade III or tumor occupying the CPA, with no displacement of the cerebral trunk, up to 30 mm; Grade IV or large tumors, with displacement of the trunk or cranial nerves, >30 mm. Even though the VS incidence has not significantly increased over the last decade, what surely has increased is the rate of small VS at diagnosis; this is mainly attributable to recent improvements in imaging techniques, particularly contrast-enhanced MRI, which is nowadays capable of detecting tumors as small as 2–3 mm [12]; this led to a trend change in VS management towards an increasingly conservative approach [13]. A recent retrospective analysis of the US Surveillance, Epidemiology, and End Results (SEER) database revealed that the rate of VS managed by means of watchful waiting has increased over time, especially concerning older patients and those with smaller tumors, and predicted that by the year 2026, half of newly diagnosed VS will be initially approached with observation alone [4].

According to the EANO guidelines, watchful waiting with serial MRI scans is the preferable option for incidental and asymptomatic VS [14]. Of note, tumor size is not among the factors dictating treatment versus observation. Of course, one must bear in mind that the larger the lesion is, the higher the probability to cause symptoms. One of the main arguments urged by those who favor observation is the fact that approximately 58 to 71% of small VS are stable in size over time, as demonstrated by Fieux et al. on an 1105 cohort of VS patients discussed in multidisciplinary meetings [15]. Also, tumor growth does not necessarily prompt switching to active treatment, in fact, failure of conservative treatment for intracanalicular VS is reported to be as low as 15% even in studies with 10-year follow-up [16].

One recent retrospective study reported that a tumor size larger than 7 mm at diagnosis was associated with an increased risk of tumor growth during observation [15]. Many reports investigated the influence of initial tumor size on the extent of tumor growth over time, however, not all of them agreed that larger tumor size at diagnosis is associated with a higher risk of growth [17] and those that do often use different cutoff sizes to define the association [18,19].

Other predictors of VS growth known from the literature include IAC filling, cystic and hemorrhagic features within the tumor, hormonal treatment, extracanalicular component greater than 20 mm, young age at diagnosis and NF-2. Most authors suggest to adopt a watchful waiting approach for small, asymptomatic lesions and switch to active treatment in case of tumor growth greater than 2–3 mm per year and/or significant worsening of symptoms. Interestingly, it has been demonstrated that the rate of post-operative complications is not significantly different when comparing patients undergoing primary surgery and those undergoing surgery due to failure of conservative management as demonstrated in an epidemiological study by Schmidt et al. [20]. On the other hand, advocates of upfront surgical treatment for small VS argue that post-operative functional outcomes are far better for small lesions with respect to larger ones. In fact, small tumor size is a well-known favorable prognostic factor for both facial nerve (FN) function and serviceable hearing preservation [11,14,21,22,23,24].

Comparative studies on the three viable management options for small VS, namely observation, radiosurgery and microsurgery, have shown similar results in terms of tumor control and FN function preservation. As far as hearing function is concerned, while it is generally agreed upon that short-term hearing preservation in small VS is better in patients undergoing a conservative management than in patients subjected to active treatment, whether it is surgery or stereotactic radiotherapy [25,26], data on long- term hearing preservation are more debatable, with some studies showing that hearing function decline is faster in the observation group after the first 2 years of follow up, while it predictably remains stable over time after surgery, as measured at 10 and 15 years [26].

sRT is a viable treatment option for small (Koos grade I and II) VS as an alternative to microsurgery and it has been suggested to be associated with a lower risk of treatment-related morbidity for small and medium-sized VS as compared to microsurgery [27,28].

Conservative management is not a viable option for large VS, nor is sRT, especially if a mass effect is present. There have been, however, reports on the outcomes of sRT for large VS that are not candidate to surgical excision. Results are variable across the literature, with tumor control rates being directly associated to tumor size. For instance, in a retrospective study by Huang and colleagues on 35 patients affected by large VS large treated by gamma knife radiosurgery, it was observed that tumor volume equal to or larger than 3 cm was a significant factor predictive of treatment failure [29].

### 3.3. Tumor Regrowth after sRT

Gamma knife stereotactic radiosurgery and fractionated stereotactic radiotherapy (sRT) have proved as valuable alternatives to microsurgical excision since the early 1990s. Nevertheless, these options have different goals: microsurgery aims at complete tumor removal, while radiation therapy aims at tumor control, and growth prevention [30]. Nakamura et al. [31] suggest a follow-up neuroimaging based on MRI contrast-enhanced (T1- and T2-weighted sequences) at 3, 6 and 12 months during the first year, every 6 months during the second year and yearly thereafter. According to Yomo et al. [32], response to radiation therapy can be classified as: (1) regression: more than 10% volume reduction; (2) stabilization: volume variation within 10%; (3) enlargement: more than 10% volume increase, not requiring further intervention; (4) failure: uncontrollable tumor growth requiring further intervention and/or appearance of disabling side effects.

In a series of 78 patients who underwent sRT for VS and were observed up to 63 months, Nakamura et al. [31] classify changes in tumor volume as: (1) temporary enlargement (41%); (2) no change or sustained regression (34%); (3) alternating enlargement and regression (13%); (4) continuous enlargement (12%). Alternating enlargement and regression can be explained by repeated extension and collapse of the cystic component of the tumor. The VS enlargement can be temporary, in this case, tumor tends to growth within the first post-radiation year, regressing spontaneously within 2 years [31].

There are three main factors involved in tumor enlargement after radiation therapy: solid expansion of the tumor, tumor necrosis and tumor cyst formation. Therefore, it is strongly recommended [30,31,33] to try to avoid reintervention during the first 2 years after radiation therapy. As a matter of fact, cranial nerves are more susceptible to surgical damage during the first year. Moreover, the interpretation of MR images in the early period after radiation therapy can be confusing.

Obviously, surgery is inevitable in case of significant complications associated with tumor growth or radiation toxicity, such as brainstem compression or hydrocephalus, as illustrated by Slattery et al. in a review regarding the House Ear Clinic experience [30]. In case of failure after radiation therapy for VS, microsurgical resection is advocated. Translabyrinthine approach is usually preferred, as the chance of preserving hearing in a patient who has undergone radiation therapy is very poor [30]. In a study by Roche et al. [34] the difficulties observed during 23 surgical resections after failed radiation therapy were analyzed, and the authors reported that tumors were more difficult to dissect in 43% of cases in comparison with size-matched naïf tumors. Severe FN and brainstem adherences were the main difficulties encountered during surgery. Moreover, lack of color change between FN and tumor contributes to make dissection harder [30]. Therefore, it is not surprising that several studies have demonstrated how cranial nerve outcomes for patients undergoing microsurgery after prior radiosurgery are relatively poor [33,35,36].

### 3.4. Planned Sub-Total Resection and Residual Tumor Management

The introduction of sRT allows the surgeon to better control tumor growth while avoiding the morbidity associated with surgery. In this scenario, treatment of large and adherent tumors can consist of surgery with the aim of at least reducing the tumor to a size suitable for sRT and removing as much tumor as can be safely removed, paying particular attention to preventing FN iatrogenic injury [37,38,39,40,41,42].

Current goals of modern treatment include both maintaining long-term tumor control and maximizing FN function and QoL. FN dysfunction after surgery, in fact, has a significant impact on patients’ QoL and more emphasis is now being placed on preserving FN function at the cost of leaving residual tumors behind [8,43,44,45,46].

The strategy of sub-total resection (STR) aims to debulk enough tumor volume to relieve symptoms caused by mass effect and provide a more favorable target size for adjuvant sRT [47]. A less frequently reported outcome is the near-total resection (NTR) where the residual tumor volume is microscopic in size [48,49]. Chen et al. [50] defined STR as when 2–5% of the tumor is left behind during surgery as noted by the surgeon and if evident on 1-yr postoperative MRI; furthermore, they defined NTR as <2% of the tumor or tumor capsule left behind during surgery, as evident from the surgeon’s subjective observation and if it is manifest or absent on 1-yr postoperative MRI. Although it has been reported that some residual tumors do not grow, progressive tumor growth is a concern in patients who have STR [37,41,51,52,53,54,55,56,57].

STR and NTR in most centers are sometimes planned event, but in most of cases they represent a decision made intraoperatively as to avoid the risk of a cranial nerve or brain stem injury. Few published studies have examined the growth rates of residual tumors left in the surgical bed. Rosenberg et al. [58] reported a postoperative growth rate of 0.35 mm/year for subtotal resection (STR) compared with 0.90 mm/year for patients who did not undergo surgical intervention, as measured on an 80 patients’ cohort during a 25-year observation. Of the residual VS, the authors reported that 68% did not grow or regressed compared with 42% in the non-surgical group. Bloch et al. [37] compared patients having undergone NTR versus STR; they reported a significant difference in recurrence, with a 3% recurrence rate in the NTR group compared with 32% in the STR group. The mean time to recurrence was 3 years. The authors concluded that STR should be avoided when possible due to the higher rate of recurrence. Syed et al. [59] described that VS recurrence was seen in 3 out of 42 treated patients (7.1%) and all had undergone an STR. No recurrence was registered in the sub-group of patients treated with NTR. The mean growth rate for these 3 cases was 0.77 mm/year. Two patients demonstrated significant regrowth within a 2-year period. The third patient showed minimal regrowth only after >8 years of follow-up. Finally, Strickland et al. [60] reported in their recent re-examination that, among patients treated with STR, regrowth was observed in 12 patients (36.3%), at an average of 23.7 months (range, 6–44 months). The NTR cohort did not experience tumor recurrence in their experience. At the time of regrowth, the tumor size increased at an average of 3.83 mm when compared with the most recent stable imaging (range, 2–8 mm). Despite the intentional STR trend, the authors stated that their philosophy is to maximize surgical resection to achieve a microscopic gross total resection whenever possible, and reserve STR or NTR for those tumors that, because of cranial nerve adherence, represent a risk for facial nerve preservation in case of further removal [60].

Concerning the functional outcomes, and focusing on the postoperative FN function, Bloch et al. [37] reported House–Brackmann score of I–II in 81% of the 79 treated patients (opered by retrosigmoid, translabirinthine and middle cranial fossa approaches), with no significant differences between NTR and STR. Starnoni et al. [61] reported in their recent meta-analysis the follow-up clinical data on FN function from eight included studies [62,63,64,65,66,67,68,69] and a random-effects pooled analysis showed an FN preservation rate (HB grade I–II) of 96.1% (95% CI 93.7–98.5%) after a combined microsurgical (STR) and sRT approach. Chen and colleagues [50] concluded that the decision to leave behind a tumor attached to the FN can be justified if the incidence and the rate of tumor regrowth are acceptably low (based on tumor site, amount of tumor left behind, tumor vascularization and age) and if there is a significant benefit in terms of FN preservation. Most authors [50,59,60,61,65,66] suggest that, given the good growth control, the FN preservation and the low number of complications, the surgical approach including STR or NTR, followed by sRT in case of documented regrowth, has an excellent clinical and functional outcome, while still achieving a tumor control rate comparable to that of total surgical resection.

### 3.5. Patient’s Age

Many authors suggest the need for personalized counselling on VS management, taking into account age, comorbidities, life expectancy, and the risk of any short- or long-term side effects [70]. Varieties of demographic factors, especially age, are linked to VS size at diagnosis and to the initial treatment plan offered. Older age is associated with smaller tumors, with an observational approach when feasible, and, when pursuing treatment, with candidacy to radiation therapy [71]. While surgery may cause sudden neurologic impairment, radiation can affect neurologic function even after many years. For this reason and in the event of a tumor regrowth or a malignant transformation, some authors recommend radiation therapy only in older patients [3,72,73,74].

**Figure 2:**
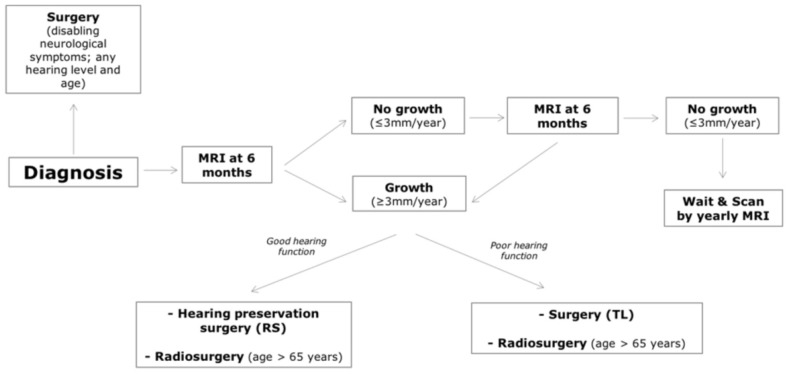
Algorithm proposed by Sergi et al. [3] to predict individualized decision-making in VS management.

In some cases, surgery is necessary even in elderly patients: the most common indications are progressive tumor growth up to large size causing brainstem compression and disabling neurological symptoms, and regrowth after previous treatment (both radiosurgery and STR). Recent evidence suggests that this population can be subdivided by age or overall health status or frailty. The concept of frailty has gained much traction in recent years as studies have identified its use to be more predictive of postoperative outcomes, compared to age alone. The analysis found that the frail group had statistically significant higher rates of readmission, postoperative infection, facial paralysis, urinary tract infection, and hydrocephalus [75]. If elderly patients are more prone to develop general complications after surgery (including acute cardiac events, stroke, bleeding, postoperative delirium, prolonged inpatient stays as well as mortality), complications specific to VS and its removal, including cerebrospinal fluid leak or FN dysfunction, were not shown to be significantly higher, as shown on a population of 452 patients divided by age cutoff > 70 years [76,77]. sRS results proved particularly favorable in elderly patients in terms of overall outcome and reliable tumor control. A study by Sergi et al. recently described an algorithm for tumor management, which considered an age cutoff > 65 years for sRT candidacy in patients with growing tumors (Figure 2) [3].

### 3.6. Symptoms

#### 3.6.1. Dizziness

Vestibular symptoms are present in 40–75% of VS patients at the time of the diagnosis [78,79], but they are rarely the presenting symptom. Compensatory mechanisms mediated by vision and contralateral vestibular apparatus probably play a crucial role in delayed presentation [80]. Vestibular symptoms in VS can be due both to vestibular nerve dysfunction and to brainstem compression, in case of tumor growth in cerebello-pontine angle. A classification system was proposed at the Consensus Meeting on Systems for Reporting Results in Acoustic Neuroma (Tokyo, Japan, 2001) [81]: grade I: normal, no dizziness; grade II: occasional and slight dizziness or disequilibrium; grade III: moderate or persistent dizziness or disequilibrium; grade IV: severe, persistent or almost persistent dizziness or disequilibrium, incapacitating and severely affecting the quality of daily life.

Andersen et al. [78], in a series of 434 patients, described how vestibular symptoms change with tumor size. Smallest tumors tend to be asymptomatic because nerve function is completely preserved. Medium-sized tumors tend to determine an increasing neuropathy, with consequent worsening dizziness and vertigo. Finally, larger tumors are associated with a more complete and stable peripheral loss, allowing better central compensation. In a study by Kentala et al. [79], in a population of 122 VS patients, 49% of patients had vertigo attacks and 69% of these attacks were mild to moderate. Moreover, this study shows how vertigo secondary to VS differs from vertigo in other peripheral diseases by the absence (63%) or low intensity (18%) of nausea. Nevertheless, there is plenty of evidence in the literature that vestibular symptoms are the most debilitating ones and have a strong negative impact on a patient’s QoL [78,80,82,83]. It is mandatory, therefore, that vestibular symptoms are taken into consideration in the therapeutic management of VS Vestibular symptoms tend to worsen when VS increases in dimensions, while seem to remain stable in case of unchanged tumor dimensions [83,84]; this indicates a favorable prognosis regarding vestibular symptoms in patients with non-growing VS. However, even in case of mild dizziness or unsteadiness, appropriate vestibular rehabilitation it is mandatory [78].

A metanalysis by Kim et al. [85] indicates that both microsurgery and radiotherapy can lead to improvement in balance outcomes in VS patients, therefore there is no significant advantage relative to vestibular symptoms between these therapeutic options. Similarly, a systematic review [86] showed that overall Dizziness Handicap Inventory (DHI) scores were not statistically influenced by intervention, irrespective of modality (surgery VS sRT). Vestibular ablation, including intratympanic gentamicin and transmastoid labyrinthectomy, could be considered in order to cope with disabling vertigo in patients with VS candidate for observation, at the possible expense of hearing loss [87]. Moreover, intratympanic gentamicin could be performed even before surgery, in order to “pre-habilitate” patients with remaining vestibular function to vestibular loss, reducing postoperative malaise and speed up recovery. The gradual reduction of vestibular function that follows gentamicin instillations allows patients to better get used to it, compared to a sudden complete loss of function as in case of surgical removal [88]. In a study by Yang et al. [87] evaluating intratympanic gentamicin treatment in patients with small VS and intractable vertigo, DHI score decreased significantly after treatment. Though patients experienced residual unsteadiness, they reported excellent relief of stress and depression related to their disabling vertigo.

#### 3.6.2. Tinnitus

The incidence of tinnitus in patients with a diagnosis of VS is reported to vary from 63% to 75%. It is the third most common symptom leading to VS diagnosis, following hearing loss and dizziness, according to a recently published survey by Peris-Celda et al. on a cohort of 1304 patients [89]. While it is clear that hearing impairment is implicated in the pathogenesis of tinnitus not associated to VS, the association of hearing impairment and tinnitus in VS patients is debated. Tumor size seems to be inversely correlated with the incidence of tinnitus and patients with larger tumor size seem to have a higher chance at tinnitus resolution after surgery [90,91].

Lately, as the focus of VS management shifts towards maintaining patients’ QoL, tinnitus has received a great deal of attention, as it is one of the symptoms with the greatest impact on psychological well-being. The presence of tinnitus should be therefore taken into account in the decision-making process [92]. In fact, there have been a number of reports claiming that active treatment, irrespective of the modality, is associated to higher rates of tinnitus improvement/resolution with respect to observation [93,94]. For instance, a study on tinnitus outcome after translabyrinthine surgery reported a statistically significant decrease in the Tinnitus Handicap Inventory (THI) score and in the Visual Analogue Scale severity of tinnitus [95]. However, these results are not consistent across the existing literature up to date. Other studies, in fact, have shown that tinnitus outcome after surgery is unpredictable, as they may stay unchanged or even worsen in a variable proportion of patients. Preservation of preoperative hearing and neurectomy of the cochlear nerve were independently associated with higher rates of tinnitus resolution after surgery in a study by Chovanec and colleagues [96]. Accordingly, Kohno et al. have reported that the prognosis for tinnitus was significantly worse in patients with anatomically preserved cochlear nerves without useful hearing than in the group with severed cochlear nerves and that severing the cochlear nerve was associated with significant efficacy in resolving tinnitus [97].

Comparisons between hearing sparing versus non-hearing sparing approaches have shown that tinnitus outcomes are better in patients treated by a translabyrintine approach than in those treated by a retrosigmoid approach, confirming the hypothesis that cochlear nerve interruption is a positive predictor of tinnitus resolution [98] Conversely, other studies deny the existence of an association between type of surgery and tinnitus resolution, suggesting that the maintenance mechanism of tinnitus is to be traced to the brainstem and central nervous system above the brainstem and not to the more peripheral structures [91,99,100]. Other factors seemingly associated with a higher likelihood of either tinnitus resolution or improvement include age > 50 years and nonserviceable preoperative hearing. Hearing preservation and cochlear nerve status did not correlate with the prognosis of postoperative tinnitus [100].

#### 3.6.3. Hearing Function

It has been shown that hearing loss in VS untreated patients can occur gradually or suddenly, regardless of tumor size [101] and that 10-years hearing preservation rate after sRT is around 23% [102]. The treatment options of VS can be divided in three main categories: observation, radiation therapy and surgery. A recent review on 3652 patients from 26 studies, and a mean follow-up of 49.2 months demonstrated consistent patterns in progression of hearing loss during observation. The authors suggest the following benchmark for those presenting with serviceable hearing (SH) at diagnosis: approximately 75% retain SH at 3 years, 60% at 5 years, and 40% at 10 years [103].

Hearing preservation after surgery varies from 18 to 82% [104]. Among surgical treatments, retrosigmoid and middle cranial fossa treatments are the only viable options for hearing preservation, without any significant differences in terms of SH preservation in the long term. A selection of patients that are candidates for surgical removal and hearing preservation is mandatory. The most commonly accepted criteria are:

- Age (usually less than 65–70 years old) [105]
- Tumor size (usually <2.5 cm, with best results when tumor <1 cm with ≥80% hearing preservation) [46,106,107].
- The preoperative hearing class, despite the size of the VS, correlates with postoperative hearing results [108].
- Degree of fundus filling: Tringali et al., using regression analysis, demonstrated that the degree of IAC involvement was the most correlated predictor of successful hearing preservation. When the fundus was completely involved, the possibility of preserving hearing dropped significantly, in absolute terms and also compared to all other degrees of IAC filling [109].

Other factors to be considered for hearing preservation treatments are patient’s general health, tumor growth pattern and characteristics, contralateral hearing, NF 2, and clinician’s experience [105]. The cornerstone in hearing preservation during surgery is the use of intraoperative monitoring techniques [46], usually ABR and/or direct eight nerve monitoring (DENM), also called CNAPs (Cochlear Nerve Action Potentials) [110]. The ABR shows some disadvantages, like being a far field technique and not having a real-time feedback on the VIII nerve status; DENM/CNAPs instead is a near field system where the electrodes are placed directly on the cochlear nerve; the signal is reproduced in a few seconds [105]. Notably, the surgeon must also be careful in preserving the labyrinthine artery, that is a terminal branch.

Despite the type of surgical approach, the highest chance of hearing preservation are shown by patients with intracanalicular VS and class A pre-opeative hearing, compared with extrameatal tumors and class B hearing or lower [11].

As far as sRT is concerned, studies report the preservation of SH in 85–87% of cases [111,112]. A recent systematic review has demonstrated differences in terms of hearing preservation comparing stereotactic radiosurgery versus fractionated radiotherapy, with slightly better results obtained by the latter (49% vs. 45% of average deterioration for patients with SH, respectively) [113]. The major bias in the reviewed articles is the length of patients’ follow up, which is usually around 5 years, not enough to anticipate 10 or 20 years behavior of SH, neither for conservative observation, nor after radiation therapy, nor after preservation surgery.

### 3.7. Multidisciplinary and Personalized Management of VS in Our Experience

In our experience, the MDT is composed of an otolaryngologist, neurosurgeon, and radiotherapist and is held once/twice a month. In a previously published paper [3], we described the results of a retrospective study on 107 consecutive patients treated by our vestibular schwannoma MDT from June 2016 to December 2019. The analysis included patient age, tumor size, hearing level, facial nerve function, tumor control, complications, and quality of life questionnaires. The median follow-up time was 30 months (range: 12–54). The median pre-treatment Koos grading was 2 (range: 1–4) and all patients had pre-treatment grade I facial nerve function. For what concerns the outcomes, according to treatment modality, in the MS group (22 patients) all subjects underwent a complete removal of the tumor; 18% of patients showed postoperative facial nerve dysfunction and no serviceable hearing (AAO-HNS Class > B) was present after surgery. In the sRT group (11 patients), one patient complained of facial paresthesia and postural instability after treatment, spontaneously resolved in 2 weeks, and one patient had posttreatment hearing worsening (class B to class D). The tumor control rate was 100%, and the mean volume reduction was 6.3 mm (range: 4–8.7 mm). Among those patients, 3 had transitioned from stage III Koos to stage II Koos, while 8 out of 22 patients had unchanged Koos stage after treatment. Finally, in the WS group (74 patients), during follow-up time hearing class worsened in 5 patients. No further symptoms occurred during observation and tumor size remained unchanged at subsequent MRI examinations in all cases.

About quality of life, we administered the Short Form-12 (SF-12), a multipurpose measure of health status composed of 2 main items: the Physical Component Summary-12 (PCS-12) score and the Mental Component Score-12 (MCS-12) score. Significant differences between groups were detected in the PCS-12 item, with higher scores in the WS group compared with the MS and sRT groups (*p* < 0.05 in both comparisons).

The review of the literature described in this paper has focused our attention on the problem of age and on the possible worst surgical outcome in elderly patients. However, a clear cutoff for age is missing, as many studies refer nonspecifically to the concept of “frailty”; this finding led us to the decision to stratify patients according to the cutoff > 65 years, based on data reported above.

## 4. Conclusions

Decision-making in VS management has progressively become more challenging. An individualized proposal of procedures is preferable as compared to a single gold-standard approach. The present review analyzed which patient and tumor’s factors need to be considered when dealing with a VS diagnosis, the most significant being age, tumor size and hearing preservation.

## Data Availability

Data are available upon reasonable request.
